# Cor Triatriatum Sinister in a 34-Year-Old Woman

**DOI:** 10.1016/j.case.2025.05.003

**Published:** 2025-07-18

**Authors:** Michael L. Cheng, Alex Gyftopoulos, Justin Brilliant, Joshua L. Leibowitz, Mark R. Vesely, James M. Brown, Manu Mysore

**Affiliations:** a Division of Cardiovascular Medicine, Department of Medicine, University of Maryland, Baltimore, Maryland; b Division of Cardiac Surgery, Department of Surgery, University of Maryland, Baltimore, Maryland

**Keywords:** Cortriatriatum sinister, Atrial mass, Echocardiography, Multi-modality imaging

## Abstract

• Cor triatriatum sinister is a rare congenital heart condition.
• Cor triatriatum rarely presents in adulthood.
• Multimodality cardiac imaging is valuable when echo findings are unclear.
• A multidisciplinary approach is needed for management of cor triatriatum.

## Graphical abstract

**Figure:**
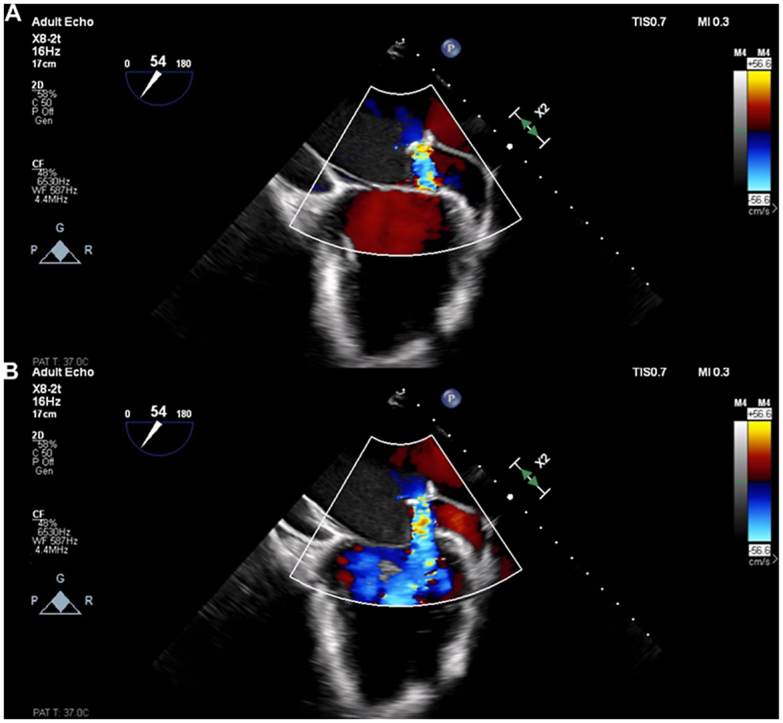

## Introduction

Cor triatriatum is a rare congenital heart condition, accounting for 0.1% of all congenital defects, in which a thin, fibromuscular membrane subdivides the left (cor triatriatum sinister [CTS]) or right (cor triatriatum dextrum) atrium into two chambers, resulting in a total of three atrial chambers. Although very rare, CTS is the most common form and results from the incomplete absorption of the common pulmonary vein. The resulting membrane may be complete or contain one or more fenestrations of differing sizes. Cor triatriatum most commonly presents in childhood, and diagnosis in adulthood is rare. Here we describe a rare case of symptomatic CTS in an adult patient.

## Case Presentation

**Figure 1:**
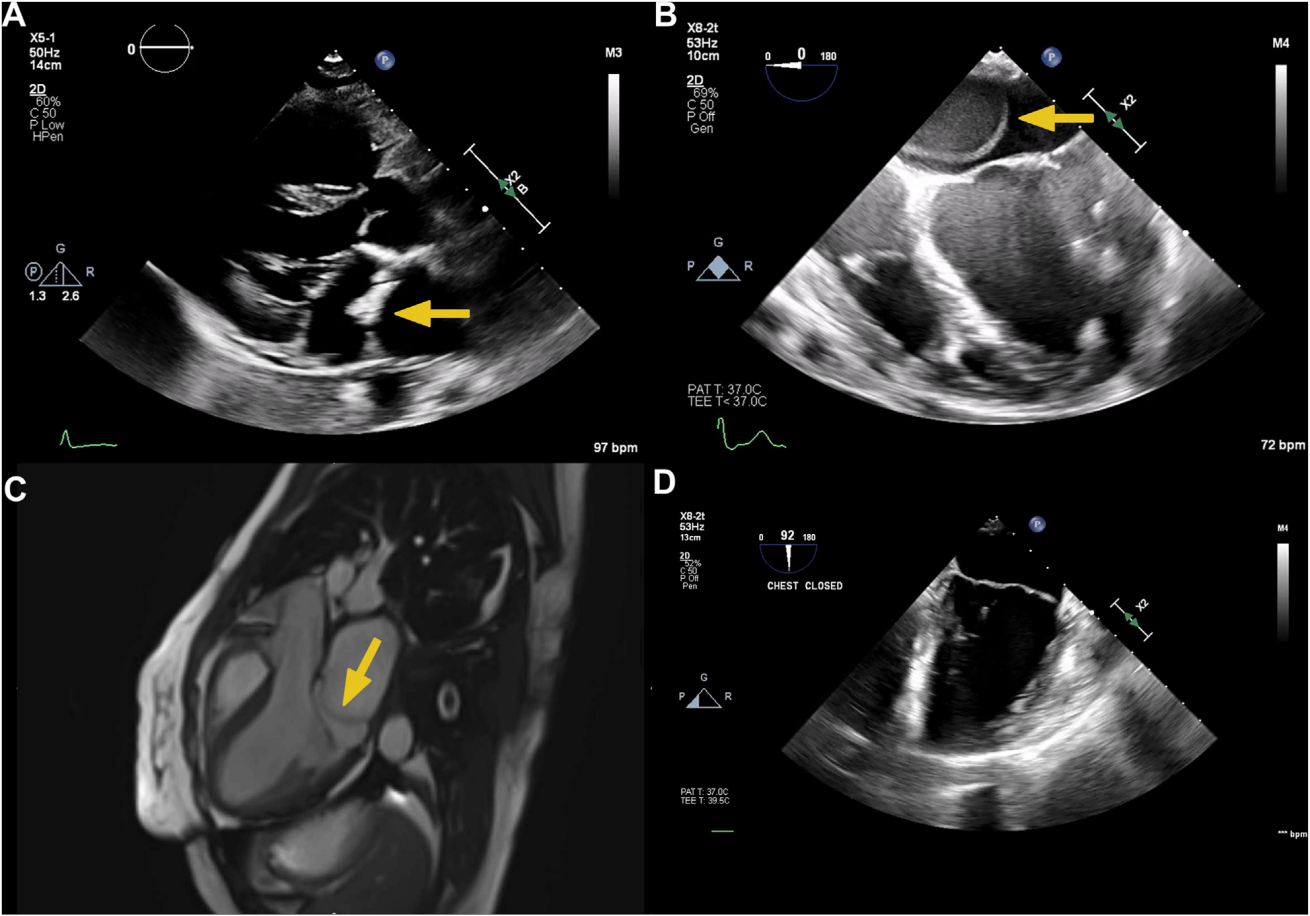
Multimodality comparison images. **(A)** Two-dimensional transthoracic echocardiography, parasternal long-axis systolic view, **(B)** two-dimensional TEE, midesophageal four-chamber (0°) low-quality (overgained), systolic view, **(C)** CMR, balanced steady-state free precession sequence, three-chamber diastolic display, and **(D)** postoperative two-dimensional TEE, midesophageal two-chamber (92°) systolic view, demonstrate the fibromuscular division of the left atrium proximal to the MV plane *(arrows)*, consistent with the CTS membrane and the normal left atrium and MV after resection.

A 34-year-old patient with no significant medical history presented with syncope and seizure-like activity. History was significant for 3 months of progressive dyspnea on exertion. The physical examination was significant for a normal S1, a normal S2, and a mid-diastolic murmur, loudest at the apex. There were no significant abnormalities on admission laboratory studies, including metabolic panel and complete blood count. Further workup included transthoracic echocardiography (Figure 1A, Video 1) which revealed a left atrial membranous structure that subdivided the left atrium. Differential diagnosis for the membrane included left atrial dissection and congenital defects such as supravalvular mitral ring (SVMR) or cor triatriatum.

**Figure 2:**
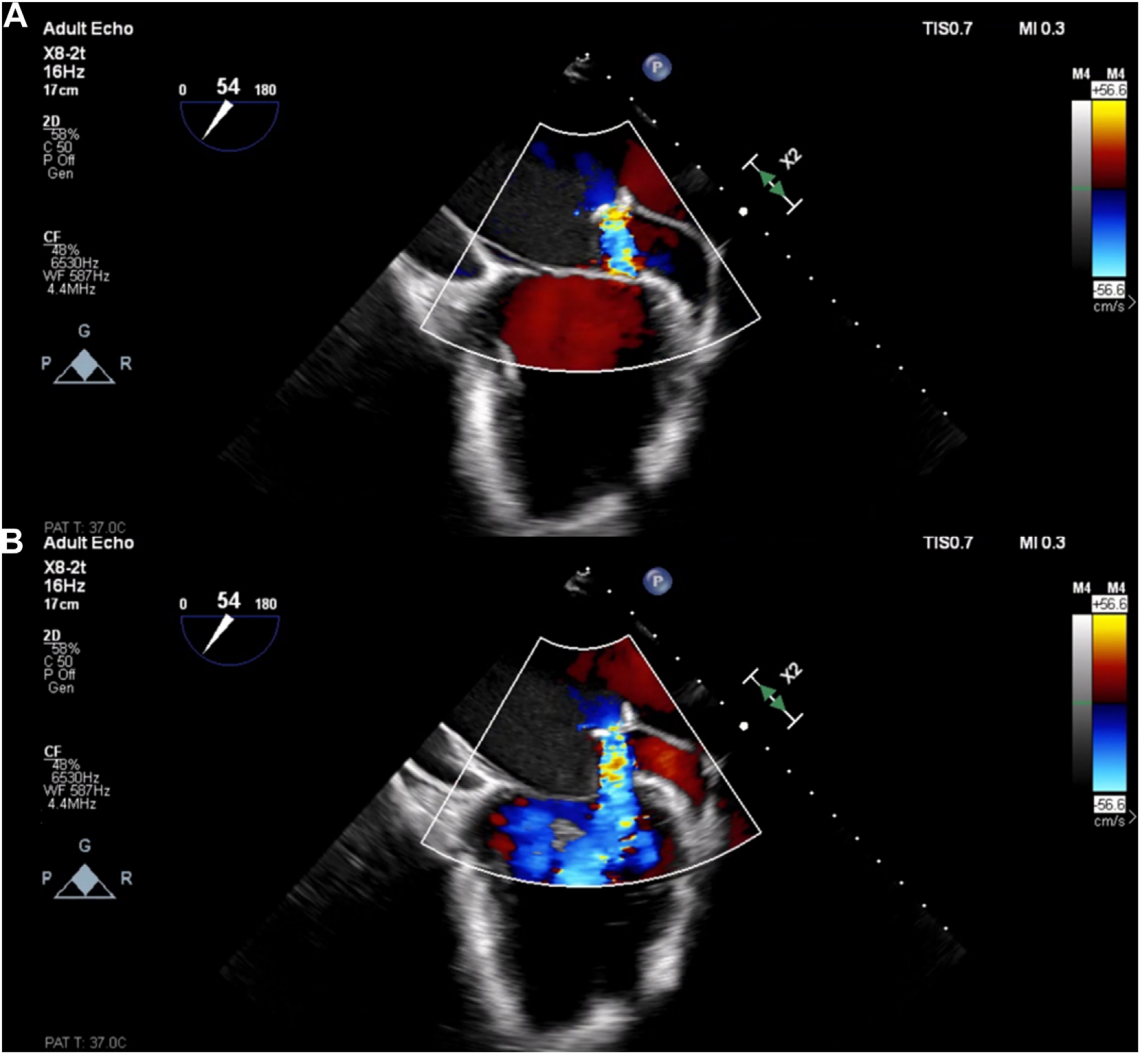
Two-dimensional TEE, midesophageal commissural view (54°), with color flow Doppler in systole **(A)** and diastole **(B)**, demonstrates the continuous flow across the cor triatriatum membrane aperture within the left atrium.

The patient underwent transesophageal echocardiography (TEE; Figures 1B and 2, Videos 2 and 3) and cardiovascular magnetic resonance (CMR; Figure 1C, Video 4), which showed a thin, fibromuscular division of the left atrium that subdivided the left atrium proximal to the mitral valve (MV) and left atrial appendage (LAA), with a singular defect at the level of the P1 scallop of the MV, consistent with CTS. Cardiac computed tomography (CCT) and CMR demonstrated normal pulmonary vein anatomy without the presence of other congenital abnormalities such as patent foramen ovale, atrial septal defect, or bicuspid aortic valve. Invasive hemodynamics, including simultaneous pulmonary artery wedge pressure and left ventricular end-diastolic pressure, demonstrated a mean gradient of 8.5 mm Hg across the membrane. Preoperative coronary angiography did not show any evidence of obstructive coronary artery disease. The patient’s dyspnea on exertion was deemed secondary to symptomatic cor triatriatum. After multidisciplinary discussion with cardiology, radiology, and cardiac surgery, the decision was made to proceed with surgical resection, which was performed successfully (Figure 1D, Video 5).

Surgery was performed via median sternotomy with bicaval cannulation for cardiopulmonary bypass. The left atrium was entered through a transseptal approach from the right atrium. Upon entry of the left atrium, all four pulmonary veins were visualized and were anatomically normal, but there was a large redundant membrane separating this atrial chamber from the MV, consistent with preoperative imaging. There was an area of calcification in the superior aspect of the membrane and a single small (8-10 mm in diameter) fenestration in the membrane. This membrane was resected flush with the left atrial wall to reveal a normal appearing MV. After closing the atria, the patient was weaned from cardiopulmonary bypass, with trace mitral regurgitation.

Regarding the patient’s presenting seizure and syncope, their inpatient neurologic workup, including magnetic resonance imaging of the brain, cerebrospinal fluid studies, and electroencephalography, was unremarkable. No clear etiology for the patient’s seizures was identified, and it was considered whether the patient had atypical syncope related to their cor triatriatum and potential left ventricular inflow obstruction, but this could not be confirmed definitively. The patient did not have recurrent seizures or syncope after initial presentation during their admission. The patient was treated empirically with the antiepileptic drug levetiracetam. On follow-up approximately 2 months after hospital discharge, the patient was doing well, with resolution of dyspnea, improvement of functional status, and no recurrence of seizures or syncope.

## Discussion

The differential diagnoses for atrial masses and membranous structures are broad, and recognition of uncommon etiologies is crucial for appropriate management and good clinical outcomes. Given the rarity of cor triatriatum, through our case we highlight the presentation, workup, and management of this condition to help expand the knowledge base for this congenital cardiac defect.

CTS most commonly presents in infancy, unless there is a sizable opening in the membrane to allow sufficient forward flow of blood. In infancy, CTS typically presents with signs and symptoms of pulmonary hypertension and pulmonary venous obstruction. Adults found to have the disease are usually asymptomatic because of the presence of a large foramen with no intra-atrial pressure gradient. Symptoms may develop because of fibrosis or calcification of the membrane foramen, creating a significant left ventricular inflow obstruction, as was demonstrated in our case by invasive hemodynamics.^1^^,^^2^

The most common presenting symptoms of cor triatriatum in adulthood include dyspnea on exertion, orthopnea, easy fatigability, and hemoptysis. Our patient was initially admitted for seizures and syncope, and their report of subacute progressive dyspnea on exertion was consistent with conventionally reported symptoms.^3^ To date, there have not been any published reports associating cor triatriatum with seizure. It was debated whether our patient had true seizure vs atypical syncope due to left ventricular inflow obstruction from the cor triatriatum, but we could not confirm definitively. Cor triatriatum presentation often mimics mitral stenosis, but the absence of a loud S1 and an opening snap on our patient’s physical examination helped distinguish between the two conditions initially.

Echocardiography remains the diagnostic imaging modality of choice for the initial evaluation of cardiac masses and membranous structures. In our case, TEE was helpful in establishing the diagnosis and providing anatomic and functional evaluation that guided surgical planning. The utility of TEE for diagnosis and surgical planning has been previously reported.^4^, ^5^, ^6^ Both CMR and CCT may be used to further define the anatomy of the defect and rule out additional cardiac anomalies and to confirm the diagnosis when echocardiography is inconclusive. Cross-sectional imaging with CCT or CMR can be particularly useful in distinguishing cor triatriatum from other types of cardiac masses and membranous structures, such as SVMR, which can have similar clinical presentations.^7^^,^^8^ In our case, cor triatriatum was distinguished from SVMR by the location of the membrane proximal to the LAA (the SVMR arises from the left atrial wall distal to the LAA). Additionally, SVMR rarely occurs as an isolated defect, and other congenital heart defects coexist in 90% of patients, which were not seen on the multimodality imaging workup of our patient.

Asymptomatic patients need no specific treatment but should be followed regularly in the outpatient setting and monitored for development of symptoms. For symptomatic patients, surgery is the definitive treatment, with a >90% survival rate over 5 years.^4^^,^^9^ Complete surgical resection of the membrane is typically performed through a midline sternotomy under bicaval cardiopulmonary bypass, as was done in the case presented here.

## Conclusion

CTS is a rare congenital heart condition that even more rarely presents during adulthood. This case highlights the importance of multimodality cardiac imaging in the diagnosis of cardiac masses and membranous structures. Early recognition of symptomatic cor triatriatum and referral to surgery are important in achieving a good clinical outcome.

## Ethics Statement

The authors declare that the work described has been carried out in accordance with The Code of Ethics of the World Medical Association (Declaration of Helsinki) for experiments involving humans.

## Consent Statement

The involved patient provided explicit verbal consent for publication of this presentation. The patient’s privacy and anonymity were protected by excluding identifiers from this case report.

## Funding Statement

The authors declare that this report did not receive any specific grant from funding agencies in the public, commercial, or not-for-profit sectors.

## Disclosure Statement

The authors report no conflict of interest.

## Supplementary Data

Video 1 Two-dimensional transthoracic echocardiography, parasternal long-axis view, demonstrates normal left ventricular size and function with a thick, membranous vertical structure seen within the left atrium.

Video 2 Two-dimensional TEE, midesophageal four-chamber (28°) view without *(left)* and with *(right)* color flow Doppler, demonstrates a low-quality image that is overgained with a membrane in the left atrium that is proximal to the MV annulus and is noted to move toward the MV during diastole, consistent with CTS; the etiology of the turbulent flow pattern is unclear.

Video 3 Two-dimensional TEE, midesophageal commissural view (54°) with color flow Doppler, demonstrates the continuous flow across the cor triatriatum membrane aperture within the left atrium.

Video 4 CMR, balanced steady-state free precession cine sequence short (three-slice) stack, three-chamber display, demonstrates a thin fibromuscular division of the left atrium proximal to the MV plane, consistent with CTS.

Video 5 Postoperative two-dimensional TEE, midesophageal two-chamber view (92°), demonstrates the normal left atrium and MV after resection of the CTS membrane.
